# Population genetics of Bull Trout (*Salvelinus confluentus*) in the upper Athabasca River basin

**DOI:** 10.1002/ece3.8110

**Published:** 2021-09-30

**Authors:** Emma K. T. Carroll, Steven M. Vamosi

**Affiliations:** ^1^ Department of Biological Sciences University of Calgary Calgary Alberta Canada

**Keywords:** Bull Trout, conservation, microsatellites, population genetics, population structure, Salvelinus

## Abstract

Freshwater ecosystems are negatively impacted by a variety of anthropogenic stressors, with concomitant elevated rates of population decline for freshwater aquatic vertebrates. Because reductions in population size and extent can negatively impact genetic diversity and gene flow, which are vital for sustained local adaptation, it is important to measure these characteristics in threatened species that may yet be rescued from extinction. Across its native range, Bull Trout (*Salvelinus confluentus*) extent and abundance are in decline due to historic overharvest, invasive non‐native species, and habitat loss. In Alberta's Eastern Slope region, populations at the range margin have progressively been lost, motivating us to better understand the amount and distribution of genetic variation in headwater habitats and some downstream sites where they continue to persist. Across this region, we sampled 431 Bull Trout from 20 sites in the Athabasca and Saskatchewan River basins and assayed 10 microsatellite loci to characterize within‐ and among‐population genetic variation. The Saskatchewan and Athabasca River basins contained similar levels of heterozygosity but were differentiated from one another. Within the Athabasca River basin, five genetically differentiated clusters were found. Despite the evidence for genetic differentiation, we did not observe significant isolation‐by‐distance patterns among these sites. Our findings of ample genetic diversity and no evidence for hybridization with non‐native Brook Trout in headwater habitats provide motivation to ameliorate downstream habitats and remove anthropogenic barriers to connectivity towards the goal of long‐term persistence of this species.

## Graphical abstract

Principal component analysis for individuals sampled in the Athabasca River basin, with 95% confidence ellipses for the five population groups identified by STRUCTURE analyses. For this dataset, differentiation among groups was best explained when using these five distinct clusters that were detected compared to either the tributary or larger regional area that the sampling site was located in.

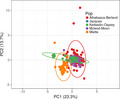

## 1 INTRODUCTION

Freshwater systems (lakes, ponds, rivers, streams, etc.) cover less than 0.01% of Earth's surface area yet harbour a disproportionate amount of global biodiversity. Estimates suggest that they support >10% of all described species, including ~30% of all vertebrate species and 40% of all fish species (Reid et al., 2020). There is mounting evidence that freshwater species are being lost at significantly higher rates than terrestrial or marine species (WWF, 2018). Indeed, the rate of population decline for freshwater vertebrates is about twofold that of terrestrial and ocean‐dwelling vertebrates (Tickner et al., 2020). Major contributors to these declines include habitat loss (i.e. destruction, degradation, fragmentation), climate change, overexploitation, invasive non‐native species, and pollution (Dudgeon et al., 2006). These factors, acting either alone or in concert, can reduce population numbers and genetic diversity, the latter of which can then further reduce long‐term population viability (Frankham, 2005; Gaggiotti, 2003).

When populations contain sufficient genetic diversity, natural selection can act on beneficial alleles to facilitate or maintain local adaption (Frankham, 2005; Tiffin & Ross‐Ibarra, 2014). Especially if habitat fragmentation is occurring, functional connectivity and gene flow between populations typically decrease, constricting the species range and increasing isolation between populations (Pierce et al., 2013). These demographic changes have genetic consequences, including population differentiation, genetic bottleneck effects and inbreeding, especially at low population densities (Neraas & Spruell, 2001). Fragmenting populations exposes the resulting sub‐populations to inbreeding effect and genetic drift, both of which further decrease genetic diversity and heterozygosity through allele fixation (Frankham, 2005). Following allele fixation, the potential for expression of deleterious recessive alleles increases, with subsequent decrease in fitness‐related traits in that population (Ruiz‐López et al., 2012). In the absence of genetic rescue via gene flow from migrants, these negative genetic consequences can lead to population extirpation (Hoglund, 2009). Quantifying and incorporating population genetic information can provide valuable guidance when attempting to identify and protect populations of a declining species (Epifanio et al., 2003; Muniz et al., 2019). Although there have been great advances in genomics of model species, our understanding of the extent and distribution of within‐ and among‐population genetic diversity remains limited for many threatened species.

Bull Trout (*Salvelinus confluentus*, Suckley 1895) is an apex predator endemic to northwestern North America. Across this vast range, spanning different watersheds, ecoregions, and management areas, the species is declining in population size and spatial extent due to overharvest, hybridization with invasive non‐native species, reduced habitat connectivity, and habitat degradation (Taylor et al., 1999). Owing to Bull Trout's important ecological role, popularity as a recreational fishery, and significance to Indigenous peoples, we have additional incentive to understand and monitor this sensitive, cold‐water dwelling species (Warnock et al., 2011). Population genetic approaches have contributed to a better understanding of how the species is responding to stressors, especially in the southern extent of their range (Ardren et al., 2011; Costello et al., 2003; Taylor et al., 1999). Bull Trout studied thus far typically exhibit hierarchical population structure (Evanno et al., 2005), whereby high levels of genetic differentiation between populations cause population substructuring within a river basin (Whiteley et al., 2004). Additionally, these patterns generally hold regardless of the presence or relative representation of different life‐history forms (i.e. resident and migratory; Homel et al., 2008). Rare gene flow between spawning streams typically results in a positive, albeit weak correlation between distance and genetic similarity, although populations typically contain unique allelic variants (Warnock et al., 2010). Because populations of threatened species typically harbour reduced genetic heterozygosity compared to not‐at‐risk congeners, it is crucial to establish presence and distribution of genetic diversity while correction measures are still viable (Spielman et al., 2004).

The Species at Risk Act (SARA) is part of Canada's strategy for protecting and managing threatened species. One important component of SARA's framework are Assessment and Status Reports, which are generated by the independent Committee on the Status of Endangered Wildlife in Canada (COSEWIC). Where there are a priori expectations for requiring management “below” the species level, genetic information can inform the establishment of Designatable Units (DUs), which represent evolutionary significant within‐species diversity (COSEWIC, 2018). These units acknowledge that unique genetic lineages and legacies are important for the evolutionary trajectory of the species. DUs are discrete, with evidence that gene flow (if present) is insufficient to overcome local adaptation. Consequently, the loss of a DU is unlikely to be reversed without deliberate intervention. At present, there are gaps in our understanding of Bull Trout in the eastern portion of its range east of the continental divide, where two putative DUs persist in allopatry: the relatively well‐studied Saskatchewan‐Nelson River population (DU4) and the understudied Western Arctic population (DU2; COSEWIC, 2012; Ripley et al., 2005). Currently, there are relatively little data on the genetic diversity or genetic relationships within and among these Bull Trout populations, especially in the headwaters region (Ripley et al., 2005; Taylor et al., 1999). With reductions to population numbers and to the overall range of Bull Trout, this gap in our knowledge of their genetics negatively affects our ability to identify vulnerable populations and adequately protect them.

Here, we assess the population genetic structure of Bull Trout, a globally recognized species as risk (COSEWIC, 2012), in the upper reaches of the Athabasca River basin. We used neutral markers to genetically identify putative populations and characterize genetic differentiation within and among populations and among river basins in Alberta. Our specific objectives were to (a) identify Bull Trout populations within the Athabasca River basin and (b) characterize within‐ and among‐population genetic variation for three major river basins (Athabasca ‐ DU4, North Saskatchewan ‐ DU2, and Bow ‐ DU2), which have evolved from the same genetic lineage (COSEWIC, 2012). Within the Athabasca River Basin, we expect that Bull Trout will follow the typical hierarchical population structuring seen in other river basins where intra‐population genetic differentiation is low, inter‐population genetic variation is high, and isolation by distance is weak. By measuring genetic diversity and differentiation, these data will help inform management decisions, such as instances where reintroductions are being considered for ameliorated sites or genetically depauperate populations suggest the existence of barriers to migration and gene flow (Dehaan et al., 2011). Overall, we provide insight into how cryptic diversity in Bull Trout may inform local management and conservation strategies.

## 2 METHODS

### 2.1 Study location and sample collection

**FIGURE 1:**
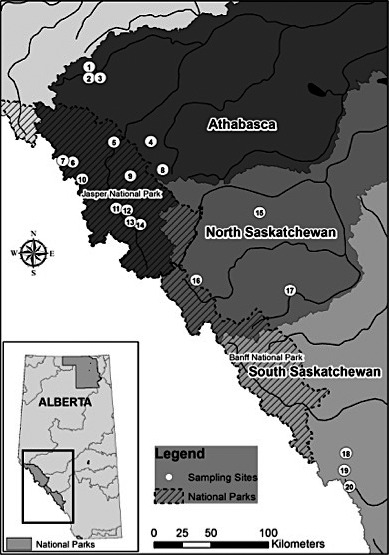
Sample sites for Bull Trout in Alberta, Canada in the Athabasca, North Saskatchewan and Bow watersheds. Colours denote different watersheds, numbers represent sampling sites (Table 1)

We sampled 14 sites to characterize the genetic diversity of Bull Trout populations within the Athabasca River Basin (Western Arctic), with six sites from the North Saskatchewan and Bow River Basins (Saskatchewan‐Nelson) sampled for comparison (Figure 1). Sites were chosen based on accessibility and known presence of Bull Trout. Both fluvial and adfluvial sites were sampled; thus individuals of different life history strategies are combined in subsequent analyses. Bull Trout ranged in fork length size from 51 mm (Berland River) to 610 mm (Athabasca River). A total of 431 individuals were captured from 2007 to 2015 at 20 locations (Table 1). Fish were caught by angling at adfluvial sites and by either backpack or boat electrofishing at fluvial sites. Upon capture, fork length measurements were taken of each individual. Adipose fin tissue samples were non‐lethally obtained by clipping and immediately transferred into 95% EtOH (Ethanol) at 4℃. Samples obtained from W. Hughson, S. Herman, M. Sullivan, and W. Warnock (Table 1) were collected using similar methods, although the latter three collectors preserved the sample by drying the adipose fin tissue and storing them in separate envelopes in a cool, dry location. All samples were collected within 8 years, a time span comparable to a single Bull Trout generation within the region. Fish collection was completed under approved fish research licences (Parks Canada: 18570 and Alberta Environment and Parks: 15‐2028) and an animal care protocol from the Life and Environmental Sciences Animal Resource Centre at the University of Calgary (AC15‐0033).

**TABLE 1:** Collection data summary

ID	Watershed	Site	Abr	Latitude	Longitude	*n*	Collector	BKTR
1	Athabasca	Berland River	BR	53.7677	−118.3523	25	Carroll■	Yes
2	Athabasca	Moon Creek	MC	53.7050	−118.3502	25	Carroll■	No
3	Athabasca	Little Berland River	LBR	53.6798	−118.2373	25	Carroll■	No
4	Athabasca	Gregg River	GR	53.2017	−117.5007	17	Carroll■	Yes
5	Athabasca	Athabasca River	AR	53.1861	−117.9821	25	Hughson■	Yes
6	Athabasca	Miette Lake	ML	53.0223	−118.5585	25	Sullivan+	No
7	Athabasca	Unnamed Lake	UL	53.0182	−118.6316	10	Sullivan+	No
8	Athabasca	McLeod River	MR	52.9847	−117.335	25	Carroll■	Yes
9	Athabasca	Jacques Lake	JL	52.9274	−117.7522	25	Carroll■	No
10	Athabasca	Derr Creek	DC	52.8853	−118.3761	1	Carroll■	Yes
11	Athabasca	Kerkeslin Creek	KC	52.6799	−117.8709	25	Carroll■	Yes
12	Athabasca	Kerkeslin Lake	KL	52.6573	−117.7875	25	Carroll■	No
13	Athabasca	Ranger Creek	RC	52.5733	−117.7160	15	Carroll■	Yes
14	Athabasca	Osprey Lake	OL	52.5592	−117.6697	25	Carroll■	No
15	North Saskatchewan	Colt Creek	CC	52.6667	−116.0689	25	Herman■	Yes
16	North Saskatchewan	Pinto Lake	PL	52.1248	−116.8637	25	Herman■	No
17	North Saskatchewan	Elk Creek	EC	52.0586	−115.6663	25	Herman■	Yes
18	Bow	Little Elbow River	LER	50.7796	−114.9537	25	Warnock•	Yes
19	Bow	Elbow River	ER	50.6748	−114.9757	13	Warnock•	Yes
20	Bow	Storm Creek	SC	50.5187	−114.9126	25	Warnock⊙	Yes

Note

Sampling years are denoted as follows: + = 2007, ⊙ = 2010, ● = 2011, ■ = 2015). Each symbol represents the different year that samples were collected since some samples were collected from government agencies in different years, the symbols provide a concise way to show that all samples were collected within one generation time of the species. Site ID and abbreviations (Abr) are used in Figure 1. BKTR denotes whether Brook Trout (BKTR) was also found in the sampled areas.

### 2.2 DNA sequence amplification

Nuclear DNA was extracted from EtOH‐preserved adipose fin tissue using a standard proteinase‐K phenol‐chloroform protocol (Sambrook et al., 1989) and stored in ddH2O at 4℃. DNA quantity (μg/μl) was determined using a Nanodrop 1000 Spectrophotometer V3.7 (Thermo Fisher Scientific), and sample concentrations were subsequently adjusted to 50 ng/μl.

During PCR, DNA regions, excised by forward fluorescently labeled oligonucleotide primers for 10 microsatellite loci (Omm1128, Sco102, Sco105, Sco106, Sco109, Sco215, Sco216, Sco220, Sfo18, and Smm22), were multiplexed in four optimized groups (Table S1). These loci were chosen based on the degree of polymorphism, ability to detect Bull Trout × Brook Trout hybrids and resolution to detect population structure of the samples (Warnock et al., 2010). Desired fragments were amplified using a C1000 Touch Thermal Cycler (Bio‐Rad, Inc.).

PCR products were run on a 1.5% w/v agarose gel using a standard gel electrophoresis protocol and visualized using a DigiDOC IT electrophoresis gel imager (UVP Inc.). Samples showing clearly defined bands representing the DNA fragment PCR products of the specified microsatellite loci were then analyzed. Fragment lengths were determined using a standard protocol for microsatellite fragment analysis with an ABI 3500XL Capillary Electrophoresis Genetic Analyzer (Applied Biosystems, Inc.) and scaled against GeneScan‐500LIZ size standard (Applied Biosystems, Inc.). For each sample, electropherograms were produced, which were scored using GENEMAPPER v.4.1 (Applied Biosystems, Inc.).

Raw genotype scores were assessed by MICROCHECKER v.2.2.3 (Van Oosterhout et al., 2004) to uncover genotyping errors and presence of null alleles that did not amplify during PCR. Because null alleles were present in no more than four of the 10 loci, all samples were used for further analyses (Van Oosterhout et al., 2004).

### 2.3 Hierarchical population structure

Among‐river basin and within– and among–sub‐basin relationships were evaluated using the Bayesian clustering method in STRUCTURE v.2.3.4 (Pritchard et al., 2000). All analyses in STRUCTURE utilized the correlated allele frequency model and flexibility in linkage disequilibrium parameters to allow the complexities of natural systems to be included into the STRUCTURE estimates (Evanno et al., 2005; Vähä et al., 2007). These parameters allow allele frequencies to be correlated between populations and are able to accurately assign individuals to closely related populations due to admixture or recent common ancestry (Falush et al., 2003). This scenario is biologically plausible in this study system due to the potential for mainstem river sampling sites to contain migratory adults from different populations that could be classified as a single population.

Including all sites within the Athabasca and Saskatchewan watersheds, 10 independent STRUCTURE runs (Evanno et al., 2005) performed at *K*‐values of 1–15 were performed to confirm genetic differentiation of Bull Trout between major river basins (Athabasca and Saskatchewan). Subsequent STRUCTURE analyses were performed on Bull Trout captured within the Athabasca River basin to determine sub‐basin structure using 10 independent runs at *K*‐values of 1–15. For all *K*‐values in STRUCTURE, 10 independent replicates were run. Initial STRUCTURE runs were conducted at 100,000 burn‐in lengths and 100,000 Markov chain Monte Carlo (MCMC) iterations (Warnock, 2008) to reveal coarse, large‐scale structure among the water basins while subsequent sub‐watershed STRUCTURE runs were conducted at 500,000 burn‐in lengths and 500,000 MCMC iterations to give more resolution to the localized population structure within watersheds (Warnock, 2008).

To determine the optimal value of *K*, the simulation summary results of the 10 independent runs for each value of *K* were evaluated with Structure Harvester v0.6.94 (Earl & vonHoldt, 2012). If multiple unique STRUCTURE plots were created at each *K*, CLUMPAK (Kopelman et al., 2015) was used to find the optimal alignment of runs at a given value of *K* using CLUMPP (Jakobsson & Rosenberg, 2007) and plotted in R version 3.4.4 (R Core Team) using the R package POPHELPER (Francis, 2017). Using the recommendations of Pritchard et al. (2000) and Evanno et al. (2005), the model in which the lowest value of *K* that encompassed the majority of the structure while also having the highest rate of change in the log probability of the data (Δ*K*) was deemed the most likely correct *K* value. The hierarchical partitioning of genetic variation among river basins and drainages using Analysis of Molecular Variance (AMOVA) in GenAlEx version 6.5 (Peakall & Smouse, 2006). The R package *adegenet*version 2.1.1 was used to perform a principal components analysis (PCA; Jombart, 2008), which corroborates these findings.

### 2.4 Population genetics

For all Bull Trout sampled from sites containing >10 individuals, departures from Hardy‐Weinberg Equilibrium (HWE), heterozygote excess and deficiency, and linkage disequilibrium (LD) between pairs of loci were determined using Markov‐chain methods and the following parameters in Genepop version 4.2 (Rousset, 2008): MCP – 100,000 dememorizations and 5,000 iterations (Warnock, 2008). Both HWE and LD tests levels of significance were adjusted using nonsequential Bonferroni corrections (Rice, 1989). Genetic diversity was calculated across all sites with genetic diversity and private alleles calculated in GenAlEx 6.5 (Peakall & Smouse, 2006) and allelic richness calculated with FSTAT version 2.9 (Goudet, 1995). Genetic divergence (*F*_ST_) and inbreeding (*F*_IS_; Weir & Cockerham, 1984) was estimated in FSTAT 2.9 (Goudet, 1995). *F*_ST_ and *F*_IS_ significance levels were adjusted using nonsequential Bonferroni correction (Rice, 1989).

Isolation by distance was tested in the Athabasca River basin using the Mantel Test to determine the relationship between pairwise genetic distance (*F*_ST_) and geographic distance (km) with 1,000 randomizations in each scenario (Dennenmoser et al., 2014; Jensen et al., 2005). Because Euclidean distance may not adequately represent realized distance between sites, we also measured the shortest waterway distance. Geographic distances in both cases were obtained using GoogleEarth (v. 4.2.1, Google Inc.).

## 3 RESULTS

### 3.1 Microsatellite loci

The degree of polymorphism displayed by the microsatellite loci of the 431 Bull Trout ranged from 7 (Sfo18) to 51 (Sco109). Genotypic frequencies differed from HWE in a number of locations and loci. Heterozygote excess was rare and found only in Little Elbow River (Sco102) and Storm Creek (Omm1128). Evidence for heterozygote deficiency was found in the populations from Colt Creek (Sco102, Sfo18), Elk Creek (Sfo18), Kerkeslin Lake (Sfo18, Sco220), Miette Lake (Sfo18), Moon Creek (Sco102, Sfo18), and Storm Creek (Sco109). One pair of loci (Jacques Lake; Sco115 and Sco220) was observed at one of 450 total locus pairs across sites to be in linkage disequilibrium after Bonferroni correction, with no linkage trends between any two loci across all sites and loci. Infrequent evidence for null alleles was seen throughout the dataset.

The two loci used to differentiate Bull Trout from Brook Trout and their hybrids, Sco216 and Sfo18, all scored alleles outside the range indicative of Brook Trout DNA, 172–192 bp and 145–165 bp, respectively (Popowich et al., 2011), corroborating that all tissues are from genetically pure Bull Trout.

### 3.2 Population genetics

Genetic diversity was not significantly different between the Athabasca River basin and Saskatchewan River basin (*H*_EAtha_ = 0.585, *H*_ESask_=0.567; Welch's *t*‐test, *t* = 0.5002, *df* = 11.149, *p* = .627). In the Athabasca River basin, expected heterozygosity values ranged from 0.473 (Gregg River) to 0.736 (Miette Lake). Allelic richness values averaged 5.52 and ranged from 2.8 (Unnamed Lake) to 8.6 (Athabasca River). Inbreeding coefficients (*F*_IS_) ranged from −0.122 (Ranger Creek) to 0.152 (Moon Creek). Of the 41 private alleles detected in this study, 20 of them were found in the Athabasca River basin, with values ranging from zero (Little Berland River and Ranger Creek) to four (Kerkeslin Creek and Unnamed Lake). Sites within the Saskatchewan River basin had similar expected heterozygosity, ranging from 0.468 (Elbow River) to 0.690 (Colt Creek). Allelic richness averaged 6.5 and ranged from 3.7 (Elbow River) to 8.1 (Colt Creek). The Saskatchewan River basin contained the remaining 21 private alleles detected, with values ranging from zero (Colt Creek, Storm Creek, and Elbow River) to nine (Little Elbow River). All calculated expected heterozygosity, private alleles, allelic richness and *F*_IS_ are presented in Table S2.

### 3.3 Hierarchical population structure

#### 3.3.1 Among‐river basin structure (broad scale)

**FIGURE 2:**
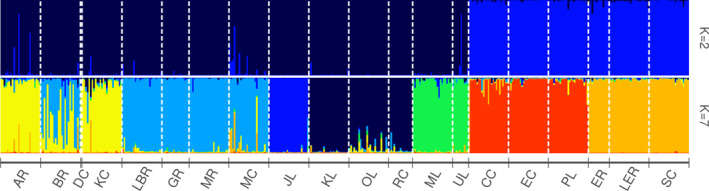
STRUCTURE result of admixture plots for Bull Trout sampled in the Athabasca and Saskatchewan River basins and sub‐basin structure within them. Admixture plots showing individual membership to *K* clusters for *K* values of 2 (above) and 7 (below) based on genotypes. Unique clusters are represented by colour. Each vertical line represents an individual. See Table 1 for sampling site codes

Genetic differentiation was detected in the Athabasca River basin and Saskatchewan River basin samples, although no differences in levels of heterozygosity were observed between the river basins. The mean *F*_ST_ value within the Athabasca River basin was 0.25, ranging from 0.05 (between Athabasca River and Kerkeslin Creek) to 0.49 (between McLeod River and Kerkeslin Lake). The mean *F*_ST_ in the Saskatchewan River basin was similar (0.24) to the Athabasca river basin samples, with *F*_ST_ values ranging from 0.15 (between Storm Creek and Little Elbow River) to 0.33 (between Elbow River and Pinto Lake) (Table 3). Using the clustering method, STRUCTURE analyses revealed a strong signal at *K* = 2 (Δ*K* = 13.36; Figure S1a,b), representing the coarsest level of structure within the area sampled. These two distinct clusters aligned with the geographical split of major river basins: one cluster contained all samples from the Athabasca River basin (Western Arctic DU4) (Sites 1–14), whereas the second cluster encompassed all samples from the Saskatchewan River basin (Saskatchewan‐Nelson DU2; North Saskatchewan: Sites 15–17; Bow: Sites 18–20; Table 1, Figures 2 and S3). Although clustered based on river basin, the majority of regional variation was explained when sampling sites were grouped based on their location within the Athabasca River basin and the Saskatchewan River basin (Table 2). Additionally, an Analysis of Molecular Variance (AMOVA) showed that across all measured regions, both among and within major river basins, the greatest amount of genetic variation was explained by population level groupings (Table 2).

**TABLE 2:** Analysis of Molecular Variance (AMOVA; Excoffier & Lischer, 2010) explained among regions (*V*_AR_), among groups (*V*_AG_), and within groups (*V*_WG_)

Source of Variation	*df*	SS	Variance	% Variance
Among regions (*V*_AR_)	1	77.34	0.14	4
Among populations (*V*_AG_)	11	408.19	0.78	22
Within populations (*V*_WG_)	571	1,459.93	2.56	74

**FIGURE 3:**
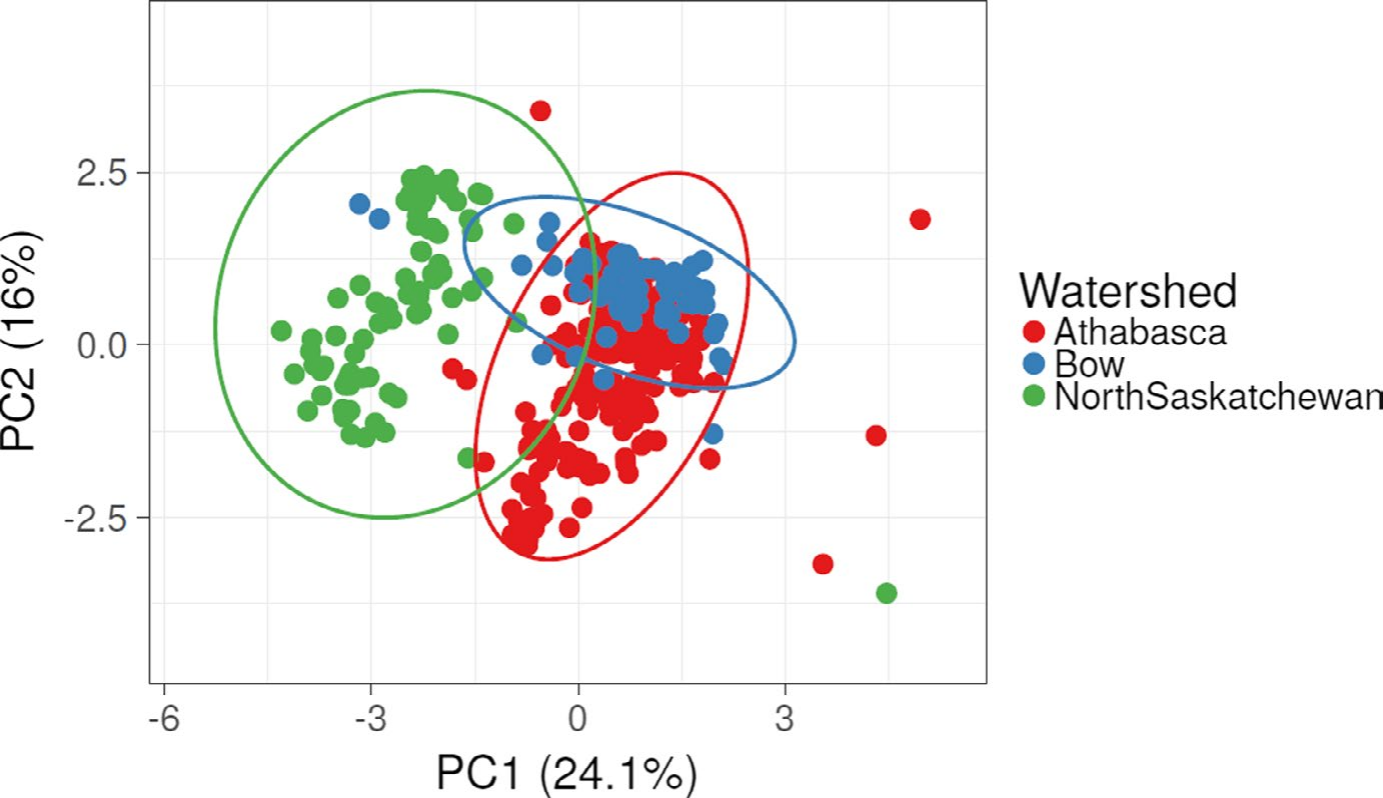
Principal components analysis for all individuals sampled in the Athabasca, Bow and North Saskatchewan River basins, with 95% confidence ellipses for each group. *N* = 431

The principal component analysis of microsatellite data from all river basins is generally consistent with evolution from a common genetic lineage (Figure 3). The first axis (PC1, 21%) largely separated some highly differentiated individuals in the Bow River Basin from all other individuals. There was additionally a weaker signal of differentiation of some individuals in the Athabasca River Basin from other individuals. The second axis (PC2, 17.9%) revealed a contrasting pattern, with marked differentiation of some Athabasca River Basin individuals and less pronounced differentiation of some individuals in the Bow River Basin. Consequently, individuals from the North Saskatchewan were least differentiated from the other river basins (Figure 3). The remaining PCA axes explained low amounts of variance in the dataset.

#### 3.3.2 Within‐river basin structure (fine scale)

Within the Athabasca River basin, further genetic differentiation revealed additional levels of structuring. Cluster‐based analyses in STRUCTURE revealed five sub‐basin archipelagos, each containing samples from one or more drainages within it. From the 14 sampling sites in this watershed, the strongest signal occurred at *K* = 5 (Δ*K* = 196.96; Figures 2 and S2a,b). These clusters aligned reasonably well with geographical drainages present in the water basin (Table 1), clustering sites from the Rocky drainage (Jacques Lake) and Miette drainage (Miette and Unnamed Lake) to their respective drainages. This supports the supposition of impassable barriers between the sampled areas relative to the mainstem Athabasca River (W. Hughson, 2015, personal communication). The three other clusters each contained fish sampled from sites spanning larger areas and different drainages. The three mixed drainage clusters were comprised of (a) the majority of sites from the Berland drainage and the main stem Athabasca drainage (Athabasca‐Berland), (b) the upper Kerkeslin drainage site with all the Ranger drainage sites (Kerkeslin‐Osprey), and (c) all McLeod drainage sites with an upper Berland drainage site (McLeod‐Moon). We note that *K* = 5 clusters accounted for more genetic variation than grouping sites by drainages (Table 2). Regardless of regional grouping, the majority of the genetic variation was explained within sampling sites.

In the North Saskatchewan River basin (Figure S4) and the Bow River basin (Figure S5), only preliminary STRUCTURE analyses were performed due to small sample sizes within river basin.

**FIGURE 4:**
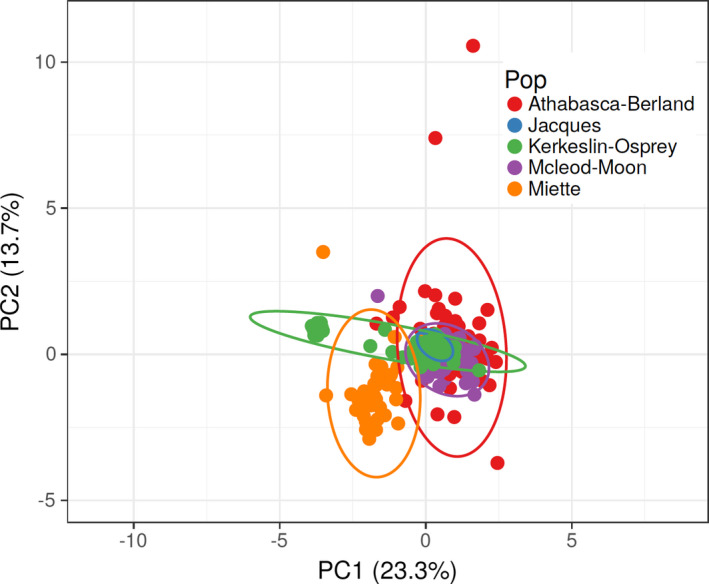
Principal components analysis for individuals sampled in the Athabasca River basin, with 95% confidence ellipses for the five population groups identified by STRUCTURE analyses (see Figure 2; *N* = 293)

The principal component analysis of populations within the Athabasca River based on microsatellite data revealed one axis (PC1, 23.3%) primarily reflecting differentiation of Miette individuals from all others but for a cluster of Kerkeslin‐Osprey individuals, which were themselves differentiated from nearly all others, including the majority of Kerkeslin‐Osprey individuals (Figure 4). On this axis, most individuals from Athabasca‐Berland, Jacques, and McLeod‐Moon were indistinguishable from each other. The second axis (PC2, 13.7%) revealed some very highly differentiated Athabasca‐Berland individuals, as well as additional, albeit less marked differentiation, of a rather large portion of Miette individuals on the other end of the axis. Collectively, Miette was the most differentiated from the other four drainages, corroborating the STRUCTURE results. Incidentally, although Jacques was not differentiated on these two axes, its 95% confidence ellipse was by far the smallest of the five drainages. As with the full dataset, the remaining PCA axes explained low amounts of variance in the dataset.

### 3.4 Isolation by distance

In the Athabasca River basin, pairwise *F*_ST_ values were variable at all geographic distances. Mantel tests performed on Athabasca River basin Bull Trout revealed no significant isolation‐by‐distance pattern between genetic distance and geographic distance using either waterway distance (*Z* = 45.78, *r* = 0.079, *p* = .29, *df* = 169) or linear distance (*Z* = 36.69, *r* = 0.072, *p* = .19, *df* = 169).

## 4 DISCUSSION

### 4.1 Overview of population structure of Bull Trout

Population genetics is an important conservation tool for understanding the genetic health of vulnerable populations (Tiffin & Ross‐Ibarra, 2014). Evaluating the status of a species' genetic diversity through population genetic techniques enables us to detect population trends, such as inbreeding depression (Restoux et al., 2012), hybridization (Rhymer & Simberloff, 1996), and population isolation by a cryptic barrier (Bergek & Björklund, 2007). In this study, Bull Trout exhibited patterns of high inter‐population genetic differentiation within river basins with the majority of variation explained at the population level, a pattern found in other Salmonids in highly fragmented systems using a similar suite of markers (Ardren et al., 2011; Dehaan & Ardren, 2005). All metrics of genetic differentiation and diversity that we considered support the conclusion that all river basins have similar levels of diversity, albeit with unique alleles and allele frequencies in each that differentiate the basins. The Athabasca River basin was found to contain several genetic clusters that did not correspond to the differences based on drainage of origin. These clusters were identified based on allele frequencies (by STRUCTURE), differentiation (*F*_ST_, *F*_IS_, and private alleles) and genetic diversity (*H_E_* and allelic richness). The majority of genetic variation was explained within each population with less variation explained among populations, and even less variation explained by the separate river basins.

### 4.2 River basin scale differentiation and diversity

Despite the finding that Bull Trout in the Athabasca and North Saskatchewan River basins have similar levels of heterozygosity (*H_E_*), STRUCTURE results showed that the two river basins are differentiated from one another, exemplified by populations being clustered based on grouping fish to their origin in the Western Arctic or Saskatchewan‐Nelson DU. In both river basins, high heterozygosity values and low *F*_IS_ were observed, alluding to large genetic differences between populations, which is common for Bull Trout systems (Costello et al., 2003). On average, the Saskatchewan River basin had higher allelic richness than the Athabasca River basin. The higher allelic richness may be a result of the former basin's closer proximity to the suspected glacial refugium (the Columbia refuge on the southern edge of the Cordilleran Ice sheet in the late Pleistocene; McPhail & Lindsey, 1986), resulting in fewer subsequent founder effects during post‐glacial dispersal than the further dispersed Athabasca River basin fish. Similarly, there was a higher incidence of private alleles in the North Saskatchewan River basin, suggesting that each population is more differentiated in terms of novel genotypes, although this may be an artefact of fewer populations sampled in this area. Because fewer sites were sampled in the North Saskatchewan and Bow River basins compared to the Athabasca River basin, our values for genetic diversity and allelic richness may be underestimated in the former two basins due to limited localized sampling in those areas.

Anticipated differentiation for Bull Trout between the Athabasca and Saskatchewan watersheds was confirmed by STRUCTURE (Figure 2). This supports the COSEWIC (2012) designation of the two river basins as separate conservation units, the Western Arctic and Saskatchewan‐Nelson, despite deriving from a common genetic lineage. Genetic differentiation is just one method used to support this designation, but it provides context for considering how Bull Trout from these DUs may differentially respond to management strategies and climate change. Previous studies using microsatellite loci have illuminated regional genetic differentiation in Bull Trout, corroborating the designation of these groupings (Spruell et al., 2003; Taylor et al., 1999). This type of differentiation on a large scale is common among fish (McPhail & Lindsey, 1986) due to different refugia, extended isolation, and limited gene flow (Avise, 2004). Because the groups have been separated for an extended period of time, evolutionary processes (e.g. genetic drift purging or fixing mutations in the two river basin's populations) have influenced the genetic differentiation and divergence of Bull Trout in these two river basins.

### 4.3 Sub‐basin structure within the Athabasca River basin

Within the Athabasca River basin, additional substructuring was found. Genetic differences between populations were high but consistent with other Bull Trout studies (Warnock et al., 2010). For this dataset, differentiation among groups was best explained when using the five distinct clusters that were detected (Table 2) compared to either the tributary or larger regional area that the sampling site was located in. For each cluster, individuals were assigned to clusters with a high proportion of their allele frequencies matching the cluster with the exception of the McLeod‐Moon cluster, which showed signs of admixture among individuals of different sampling site origin (specifically Moon Creek and McLeod River). Within the Athabasca River basin alone, these five groups likely constitute an intermediate level within a hierarchical pattern of genetic diversity, with the majority of genetic variation explained within each of the five clusters with less variation among groupings and even less by river basins. Given that much genetic variation is explained in the population level and the McLeod‐Moon cluster (and, to a lesser extent, the Athabasca‐Berland cluster) shows signs of two different genotypes within its membership, it is possible that further substructuring may exist within these groups. One limitation of our data is that we are unable to assign migratory versus resident status to individuals. It appears likely that some of the individuals showing signs of admixture had a parent (or grandparent(s)) born in a different population, given that pairs of sites showing these patterns (such as KC and OL) tend to be spatially clustered and are either currently or historically connected. We anticipate examining the contribution of life history variation to genetic connectivity among Bull Trout populations in the region in future studies.

### 4.4 Isolation by distance

Despite the evidence of substructuring in the Athabasca River basin, no evidence for isolation by distance was found. This result suggests that the observed genetic differences are at least partly influenced by physical barriers or other unmeasured natural occurrences that impede or facilitate movement between populations in ways that do not correlate linearly with geographical distance (Slatkin, 1993). In lower, more homogenous stretches of the Athabasca River basin, another Salmonid, Arctic Grayling (*Thymallus arcticus*, Pallas 1776) exhibits moderate isolation‐by‐distance patterns, which is thought to be due to a combination of their large geographic ranges and population sizes (Reilly et al., 2014). Mountain Whitefish (*Prosopium williamsoni*, Girard 1856), also a Salmonid, tends to exhibit weak isolation‐by‐distance trends, although this has been attributed to large population sizes and high levels of gene flow, which prevent differentiation (Whiteley et al., 2006).

### 4.5 Implications

Because the range of Bull Trout is declining, this elevates the importance of conserving genetic diversity in remaining extant populations as a means to withstand stochastic events (Rieman & McIntyre, 1995) and a source for adaptation in the future (Frankham, 2005). Shrinking populations on the periphery of the range are especially at risk of extirpation as a result of isolation and habitat fragmentation (Rieman & Myers, 1997). At present, many Bull Trout populations are listed as “data deficient” with little insight into regionally specific differences from the highly studied areas from which management plans were created (COSEWIC, 2012). Establishing baseline levels of genetic diversity allows for comparisons and detection of change in future stocks in addition to evaluation of adaptive management efforts (Epifanio et al., 2003). Uncovering areas that are genetically distinct from one another and determining the level of differentiation in local river basins can guide management strategies that work at local scales. In Alberta, the genetic integrity of fish species and populations is determined based on the degree of hybridization, genetic similarity to original stock, and genetic distinction (Rodtka, 2009). In this study, we provide baseline genetic information from which to track genetic changes observed in Bull Trout populations in the Athabasca River basin. Our results suggest little hybridization with Brook Trout at present in this region, as no hybrids or backcrosses were observed or genetically detected. With regard to genetic distinctiveness, we found genetic substructuring within the Athabasca River basin. High pairwise *F*_ST_ values between sites indicate differentiation among groups (Table 3). However, high heterozygosity (*H_E_*) values and AMOVA results indicate that genetic variance largely resides within the population level. Thus, although there is differentiation between groups, there currently appears to be sufficient genetic diversity that can be drawn upon, acting as evolutionary potential to allow adaptation to changing habitat conditions and long‐term persistence.

**TABLE 3:** Pairwise *F*_ST_ estimates (Weir & Cockerham, 1984) among sample sites in lower diagonal

Site Codes	AR	BR	CC	EC	ER	GR	JL	KC	KL	LER	LBR	MR	ML	MC	OL	PL	RC	SC	UL
AR	—	283.7	—	—	—	247.5	45.0	72.2	78.9	—	280.5	283.0	91.1	293.0	98.0	—	93.8	—	89.4
BR	0.06	—	—	—	—	255.2	328.7	355.9	362.6	—	18.3	290.7	374.8	9.3	381.7	—	377.5	—	373.1
CC	0.20	0.23	—	—	—	—	—	—	—	—	—	—	—	—	—	—	—	—	—
EC	0.27	0.31	0.18	—	—	—	—	—	—	—	—	—	—	—	—	—	—	—	—
ER	0.30	0.32	0.26	0.33	—	—	—	—	—	—	—	—	—	—	—	—	—	—	—
GR	0.17	0.16	0.25	0.38	0.42	—	292.5	319.7	326.4	—	254.5	76.1	338.6	264.5	345.5	—	341.3	—	336.9
JL	0.23	0.21	0.31	0.37	0.43	0.27	—	107.2	113.9	—	325.6	328.0	126.1	338.0	133.0	—	128.8	—	124.4
KC	0.06	0.07	0.19	0.26	0.29	0.19	0.22	—	6.7	—	352.8	355.2	77.3	365.2	25.8	—	21.6	—	75.5
KL	0.36	0.39	0.33	0.33	0.48	0.46	0.45	0.30	—	—	359.5	361.9	84.0	371.9	32.5	—	28.3	—	82.3
LEB	0.27	0.31	0.25	0.29	0.17	0.37	0.39	0.27	0.45	—	—	—	—	—	—	—	—	—	—
LBR	0.10	0.06	0.26	0.36	0.38	0.19	0.30	0.13	0.45	0.34	—	290.0	371.6	27.6	378.6	—	374.4	—	369.9
MR	0.13	0.12	0.30	0.39	0.44	0.21	0.33	0.16	0.49	0.38	0.11	—	374.1	300.0	381.0	—	376.8	—	372.4
ML	0.27	0.31	0.28	0.32	0.39	0.35	0.42	0.27	0.47	0.31	0.30	0.32	—	384.1	103.1	—	98.92	—	4.1
MC	0.09	0.08	0.21	0.30	0.29	0.13	0.26	0.12	0.41	0.25	0.08	0.14	0.27	—	391.0	—	386.8	—	382.4
OL	0.15	0.18	0.25	0.35	0.38	0.23	0.32	0.13	0.35	0.33	0.19	0.23	0.31	0.17	—	—	4.2	—	101.4
PL	0.26	0.28	0.22	0.24	0.33	0.35	0.33	0.24	0.42	0.31	0.35	0.37	0.34	0.29	0.34	—	—	—	—
RC	0.24	0.22	0.29	0.36	0.40	0.30	0.35	0.18	0.41	0.38	0.28	0.34	0.40	0.20	0.17	0.35	—	—	97.15
SC	0.27	0.31	0.23	0.25	0.18	0.36	0.37	0.26	0.38	0.15	0.37	0.41	0.35	0.28	0.34	0.28	0.35	—	—
UL	0.27	0.30	0.24	0.32	0.34	0.36	0.44	0.26	0.48	0.30	0.32	0.37	0.24	0.25	0.31	0.31	0.36	0.34	—

Note

All sites pairs are significantly different from panmixia (*p* < 0.0003). Waterway distance (km) in upper diagonal for Athabasca sites. See population codes in Table 1.

## CONFLICT OF INTEREST

The authors have no conflicts of interest to declare.

## AUTHOR CONTRIBUTIONS

**Emma K. T. Carroll:** Conceptualization (equal); Data curation (equal); Formal analysis (equal); Funding acquisition (supporting); Investigation (lead); Methodology (lead); Project administration (equal); Writing‐original draft (lead); Writing‐review & editing (equal). **Steven M. Vamosi:** Conceptualization (equal); Data curation (equal); Formal analysis (equal); Funding acquisition (lead); Investigation (supporting); Methodology (supporting); Project administration (supporting); Writing‐original draft (supporting); Writing‐review & editing (equal).

## Supporting information

Figure S1a

Figure S1b

Figure S2a

Figure S2b

Figure S3

Figure S4

Figure S5

Figure S1‐5

Table S1

Table S2

## Data Availability

Microsatellite data and tables not shown available at https://doi.org/10.5061/dryad.zkh1893b9.
