# Supplementary figures and images for: Population genetics of Bull Trout (Salvelinus confluentus) in the upper Athabasca River basin
Source: Ecol Evol. 2021 Sep 30;11(21):14509–20. doi: 10.1002/ece3.8110 (PMC8571605; doi:10.1002/ece3.8110)

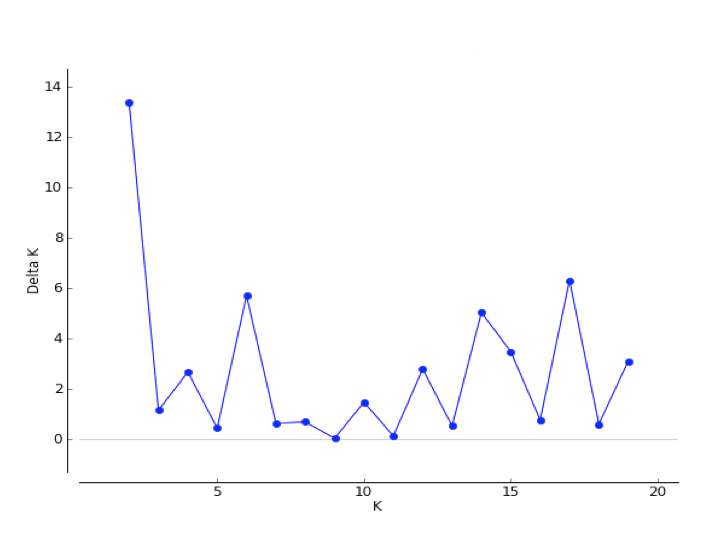

Supplement: Supplementary file 1 — Figure S1a [file ECE3-11-14509-s008.tif]

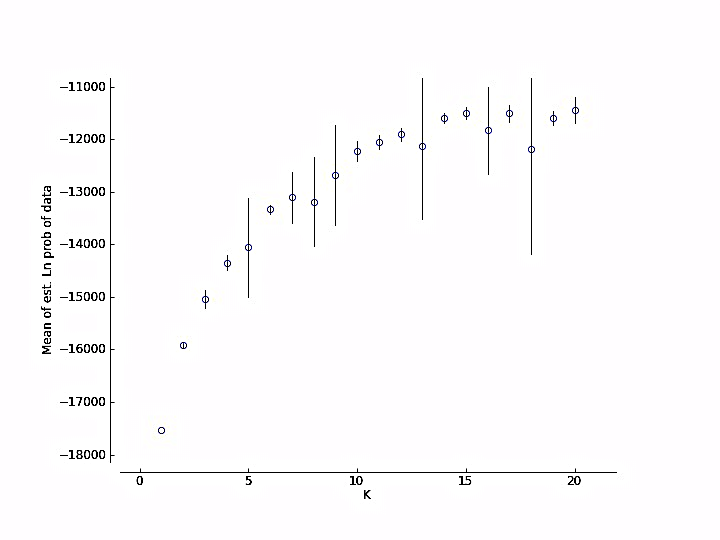

Supplement: Supplementary file 2 — Figure S1b [file ECE3-11-14509-s006.tif]

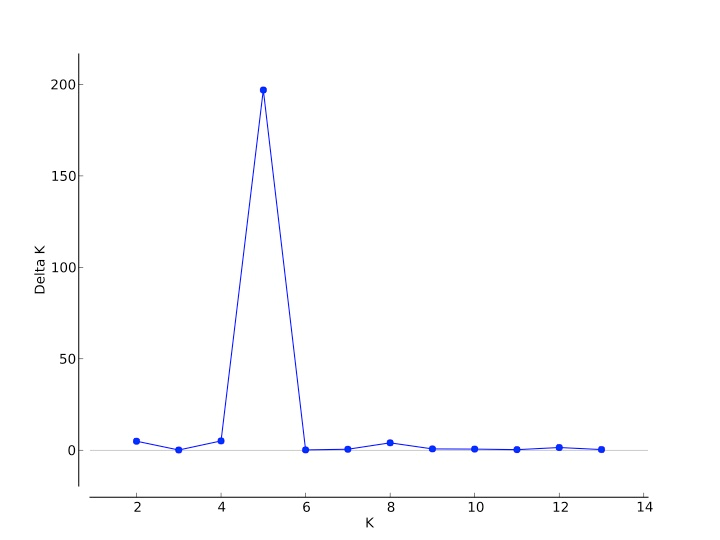

Supplement: Supplementary file 3 — Figure S2a [file ECE3-11-14509-s010.tif]

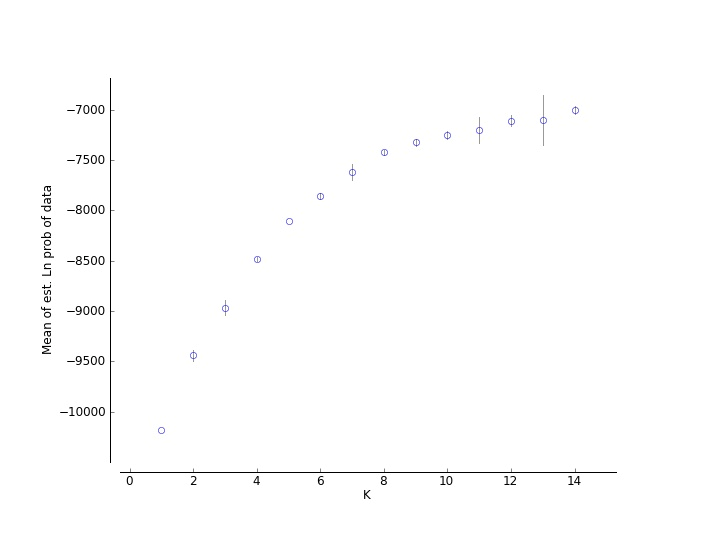

Supplement: Supplementary file 4 — Figure S2b [file ECE3-11-14509-s001.tif]

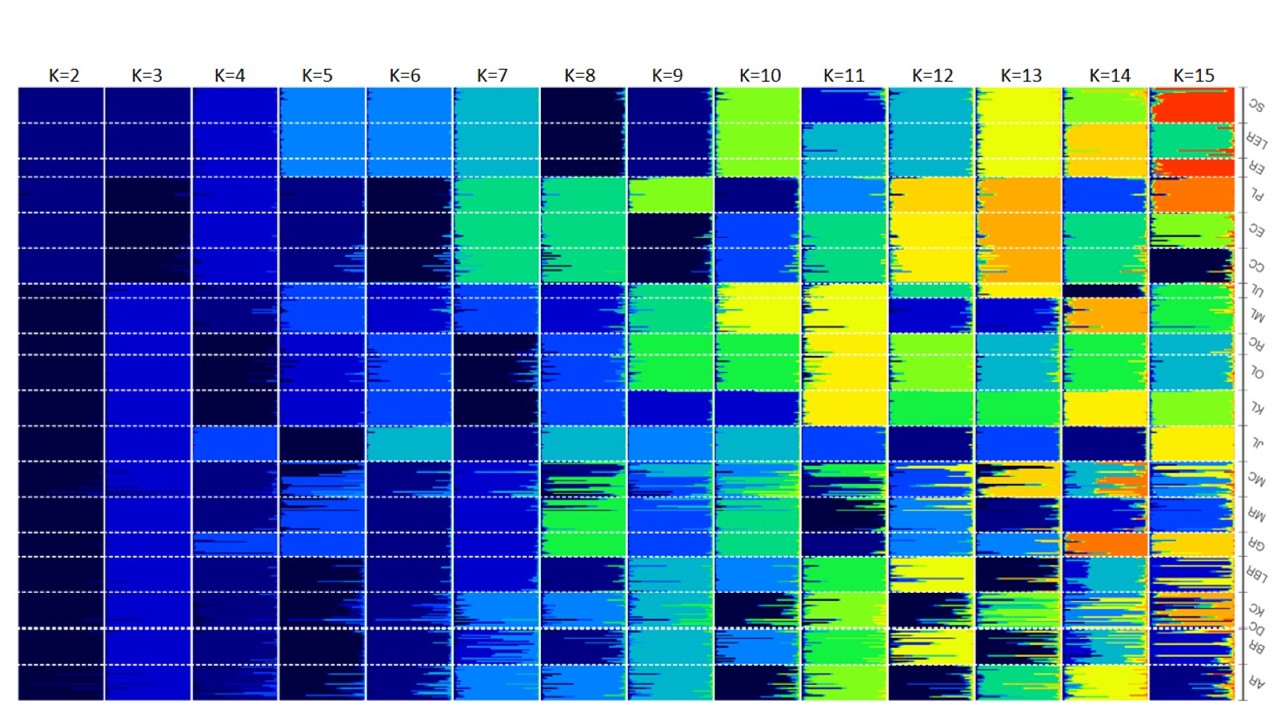

Supplement: Supplementary file 5 — Figure S3 [file ECE3-11-14509-s002.jpg]

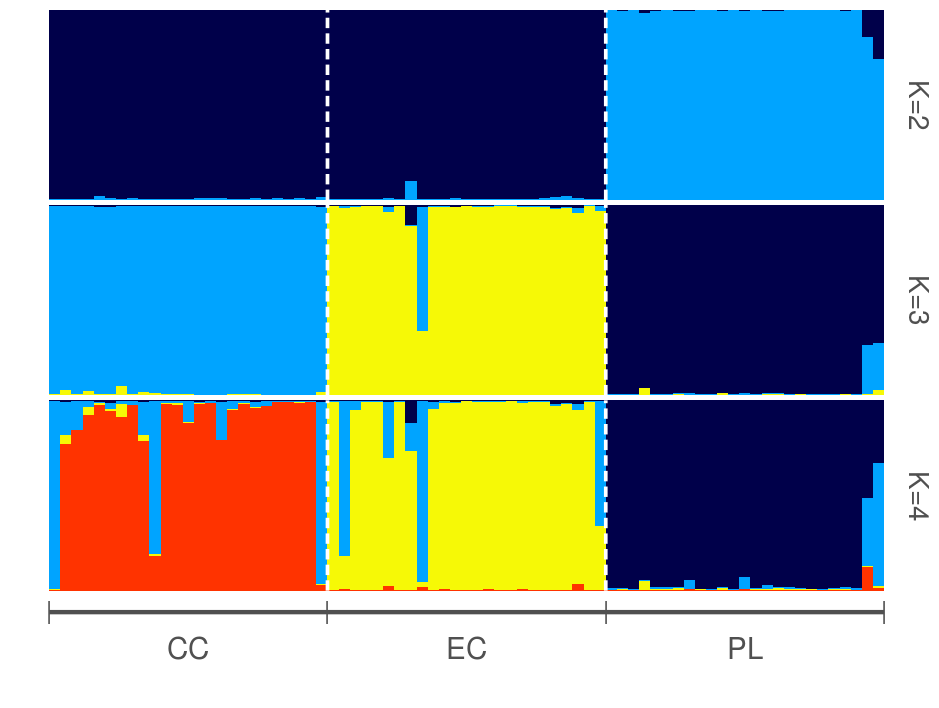

Supplement: Supplementary file 6 — Figure S4 [file ECE3-11-14509-s003.tif]

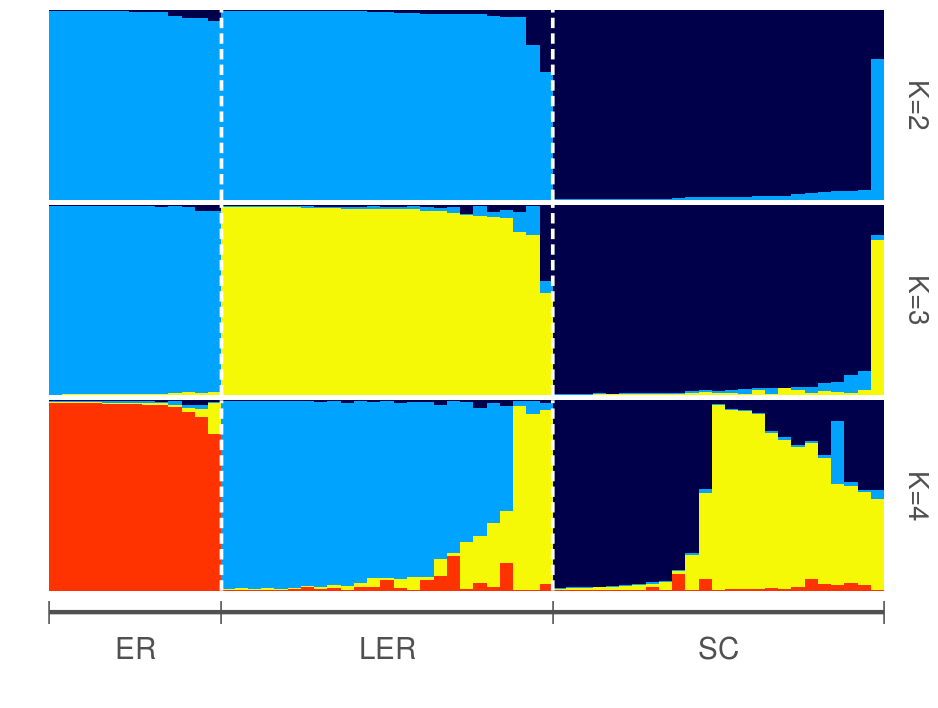

Supplement: Supplementary file 7 — Figure S5 [file ECE3-11-14509-s009.tif]

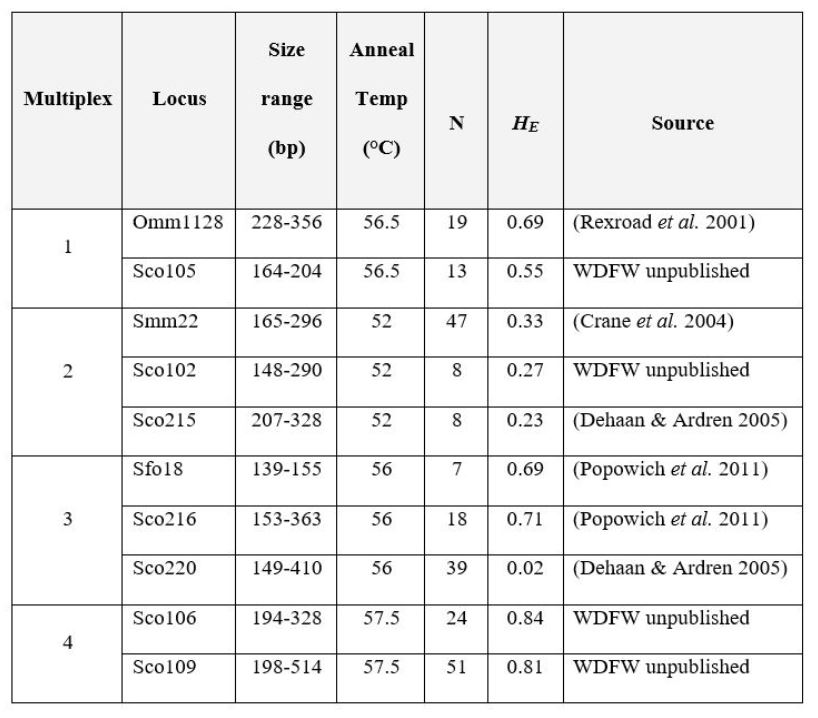

Supplement: Supplementary file 9 — Table S1 [file ECE3-11-14509-s007.JPG]

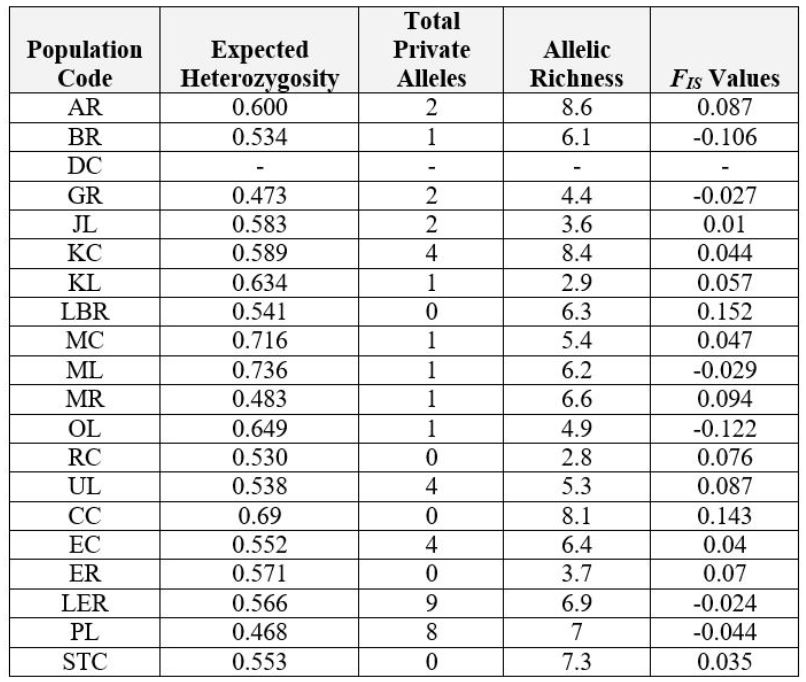

Supplement: Supplementary file 10 — Table S2 [file ECE3-11-14509-s005.JPG]
