# Supplementary material for: Population genetics of Bull Trout (Salvelinus confluentus) in the upper Athabasca River basin
Source: Ecol Evol. 2021 Sep 30;11(21):14509–20. doi: 10.1002/ece3.8110 (PMC8571605; doi:10.1002/ece3.8110)
Supplement: Supplementary file 8 — Figure S1‐5 [file ECE3-11-14509-s004.docx]

**Supplemental Figures for Appendix**

(A)


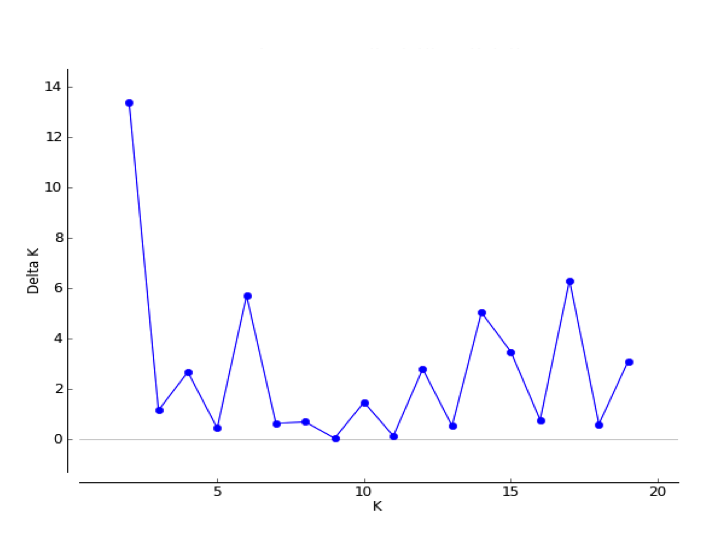


(B)


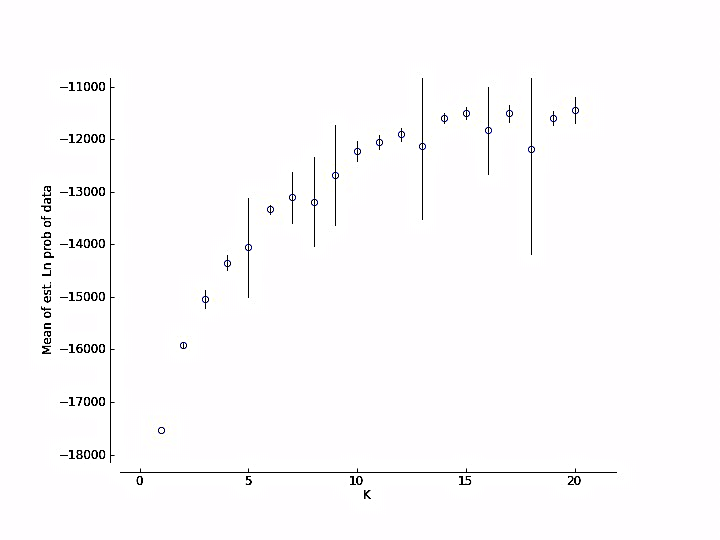


**Figure 1:** (A) STRUCTURE results of a distribution of *DeltaK* (based on Evanno *et al.* 2005) for all 20 sites sampled across the Athabasca and Saskatchewan River basins. *K* represents the number of clusters detected in the data set. (B) Posterior probability (LnP(D)) per cluster (*K*) as recommeded by Pritchard et al. (2000) with standard error bars for all 20 sites sampled across the Athabasca and Saskatchewan River basins.

(A)

**
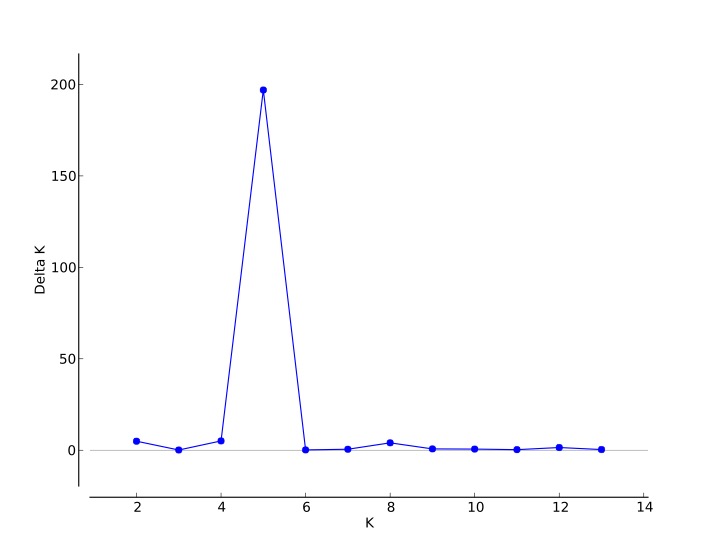
**

(B)

**
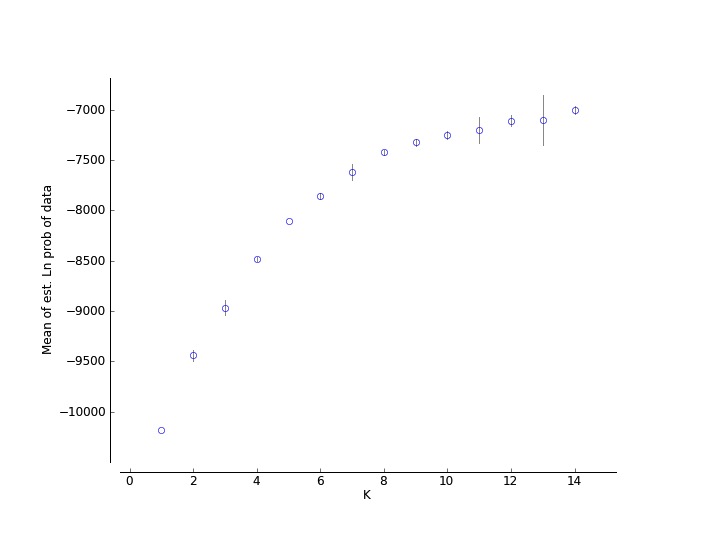
**

**Figure 2**: (A) STRUCTURE results of a distribution of *DeltaK* (based on Evanno *et al.* 2005) for only the 14 sites sampled across the Athabasca River basins. *K* represents the number of clusters detected in the data set. (B) Posterior probability (LnP(D)) per cluster (*K*; Pritchard et al. 2000) with standard error bars for all 14 sites sampled across the Athabsca River Basin.

**
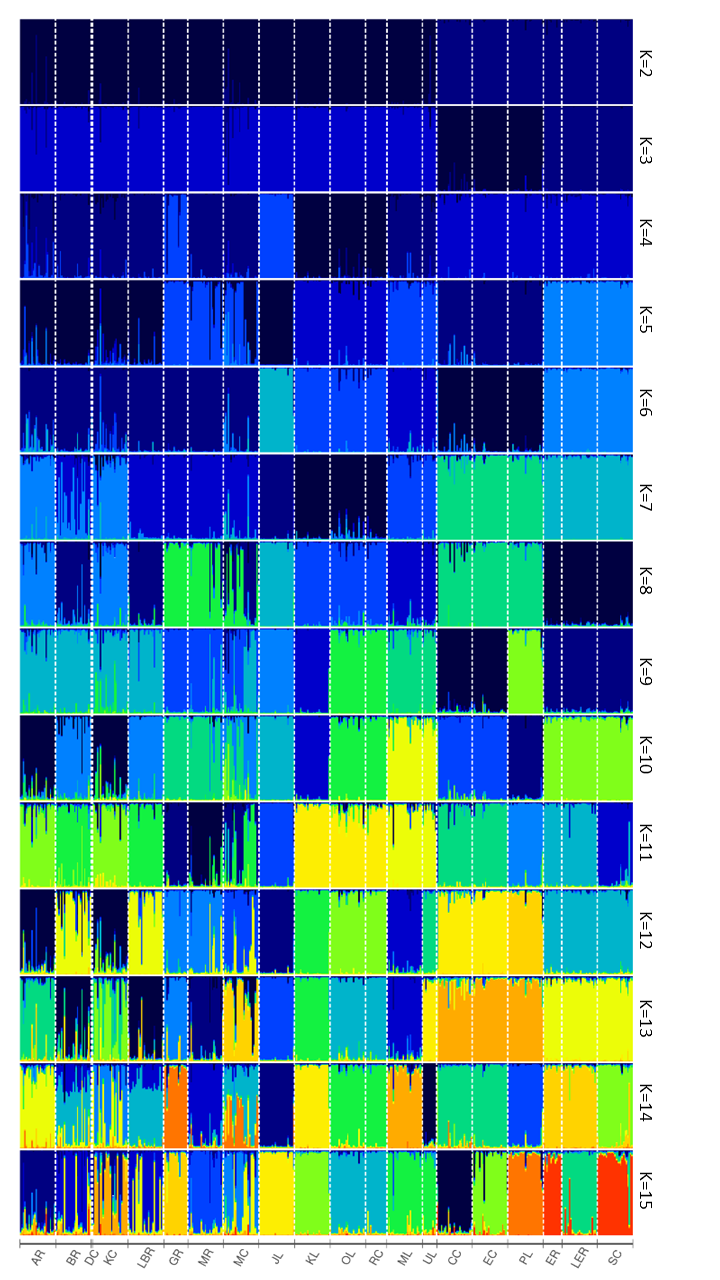
**

**Figure** 3: STRUCTURE results of admixture plots for Bull Trout sampled in the Athabasca and Saskatchewan River basins where K=2 through K=15.

**
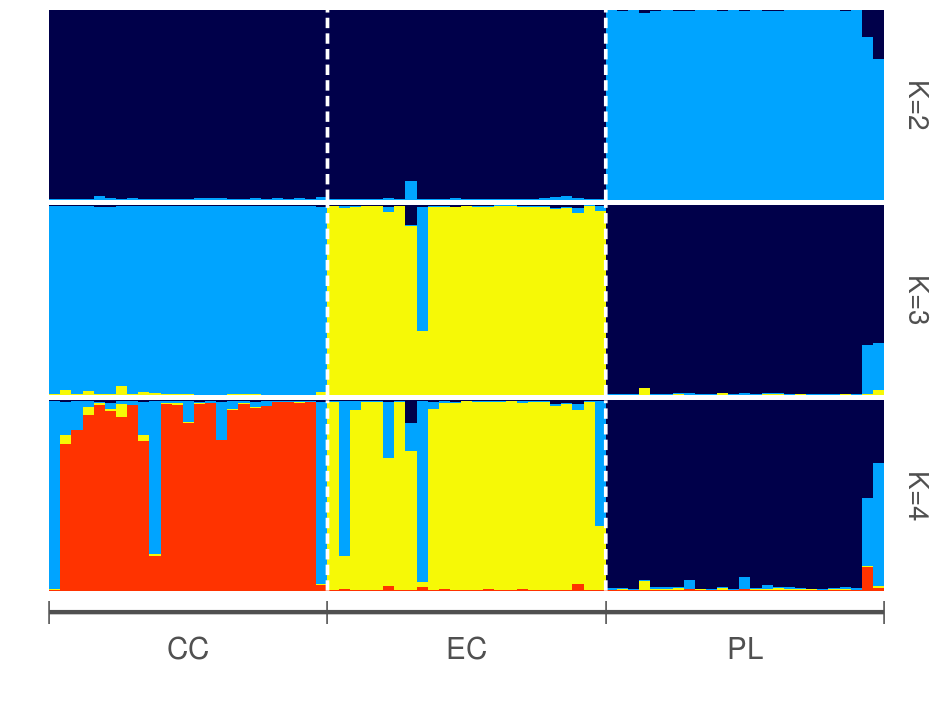
**

**Figure 4:** STRUCTURE results of admixture plots for Bull Trout sampled in the North Saskatchewan River basin where K=2 through K=4.


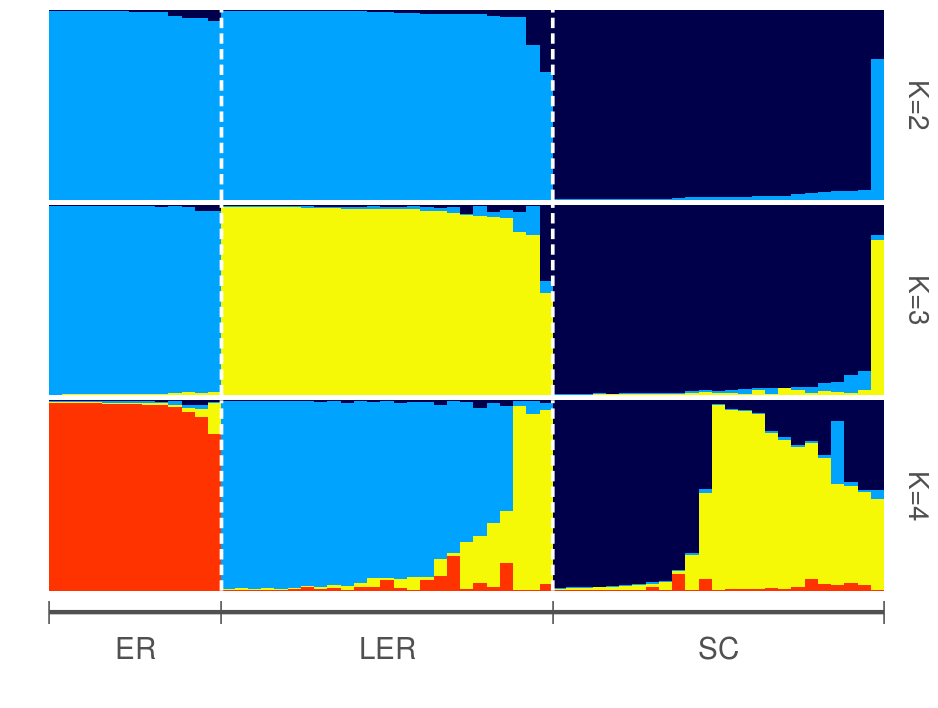


**Figure 5:** STRUCTURE results of admixture plots for Bull Trout sampled in the Bow River basin where K=2 through K=4.
